# VAT/GST harmonisation challenges for digital assets such as bitcoin and NFTs in the EU following Case C-264/14 (Skatteverket v David Hedqist)

**DOI:** 10.1365/s43439-024-00124-2

**Published:** 2024-07-02

**Authors:** Stephanie Ness

**Affiliations:** Diplomatic Academy of Vienna (Diplomatische Akademie), University of Vienna (Universität Wien), Vienna, Austria

**Keywords:** VAT/GST Compliance, EU Digital Single Market, Cryptocurrencies, Non-Fungible Tokens (NFTs), EU Tax Policy, Skatteverket v David Hedqist Case (Case C-264/14)

## Abstract

The verdict in the case of *Skatteverket v. David Hedqvist *(Kokott, Advocate General (2015) Opinion delivered on 16 July 2015, Case C-264/14. ECLI:EU:C:2015:498. Available via TandF Online. 10.1080/20488432.2015.1096631.) is crucial for understanding how the EU treats virtual currencies, such as Bitcoin, in terms of Value-added Tax (VAT). This case involved the Swedish citizen David Hedqist who was seeking clarity from the Swedish Tax Authority Skatteverket on exchanging money for Bitcoins. The case set a precedent exempting such services from VAT under the EU’s VAT Directive (Council Directive 2006/112/EC (2006) On the common system of value added tax. OJ L347. Available via EUR-Lex. https://eur-lex.europa.eu/legal-content/EN/ALL/?uri=CELEX:32006L0112. Accessed 3 January 2024.). Specifically, Article 135(1)(e) of the EU’s VAT Directive excludes those transactions from VAT that include money-related transactions, that include deals or negotiations about different kinds of money, including cash and coins that are officially legal tender, i.e., used for buying things, except for collectable items like special coins or notes that people collect but do not use as a means of payment. *Skatteverket* (Kokott, Advocate General (2015) Opinion delivered on 16 July 2015, Case C-264/14. ECLI:EU:C:2015:498. Available via TandF Online. 10.1080/20488432.2015.1096631.) clarified that cash transactions are not subject to VAT, even though they are considered services for VAT purposes.

Despite this clarity, the evolving landscape of digital assets’ uniqueness, including Non-Fungible Tokens (Alawadhi KM, Alshamali N (2022) NFTs Emergence in Financial Markets and their Correlation with DeFis and Cryptocurrencies. Applied Economics and Finance 9:108. 10.11114/aef.v9i1.5444. Available at CORE. https://core.ac.uk/download/pdf/524752899.pdf. Accessed 3 January 2024.), continues to challenge VAT frameworks across member states.

Using insights from the European Commission’s Working Paper 1060, this article advocates for a unified approach tailored to digital and crypto services, addressing complexities in NFT taxation to reduce uncertainty and foster market cohesion. The findings highlight the importance of legislative changes and increased cross-border collaboration, as well as provide recommendations for policymakers and stakeholders in the digital finance and platform sector (European Commission (2024) Working Paper 1060. Available at: https://ec.europa.eu/info/publications/working-paper-1060_en. Accessed 3 March 2024.). By proposing strategic harmonisation of VAT enforcement, the research helps to improve tax compliance and support long-term growth in the EU’s digital market (Cappai M (2023) The role of private and public regulation in the case study of crypto-assets: The Italian move towards participatory regulation. Computer Law & Security Review 49:105831. Available at: https://www.sciencedirect.com/journal/computer-law-and-security-review/vol/49/suppl/C.; Hasa J (2021) Digitaalisten palvelujen rajat ylittävä kuluttajakauppa ja laajeneva arvonlisäveron erityisjärjestelmä. Licentiate thesis. University of Lapland, Faculty of Law. Available at: https://lauda.ulapland.fi/bitstream/handle/10024/64771/Hasa_Juho.pdf?sequence=1. Accessed 1 March 2024.).

## Introduction: the challenge of taxing digital assets

In the fast-changing landscape of the digital gig and platform economy, taxing digital assets and services, notably cryptocurrencies and Non-Fungible Tokens (NFTs), poses a complex challenge to regulatory regimes around the world: NFTs (Non-Fungible Tokens) and cryptocurrencies are both digital assets operated by blockchain technology, but they differ fundamentally in their uniqueness and interoperability. NFTs are one-of-a-kind digital assets that may consist of artwork, music, or any other form of digital content. Due to their unique qualities, they cannot be replaced, which means that they are “non-fungible”. Cryptocurrencies, such as Bitcoin, are identical units that are supposed to be interchangeable or “fungible”, like traditional money in that each unit is the same as the next. While NFTs emphasise the ownership and uniqueness of specific digital assets, cryptocurrencies enable decentralised financial transactions^1^.

Exponential growth in Non-Fungible Tokens (NFTs) from digital art to real-world asset tokenization and cryptocurrencies has changed digital commerce since the ECJ’s October 22, 2015, verdict in *Skatteverket v David Hedqvist *(Case C-264/14)^2^. This change in digital commerce includes regulatory frameworks, taxation models, financial market integration, cross-border transactions, intellectual property rights, mainstream adoption, security standards and smart contracts^3^.

The sale of Beeple’s NFT in March 2021 drew global interest and showcased the ability of NFTs to transform the art industry by introducing innovative methods for dividing ownership^4^. Beyond the art world, NFTs have an impact on real estate, where they are being employed in novel ways to fractionalize ownership. This enables investors to buy and sell high-value assets with unparalleled simplicity. A pioneering example of this occurred in a transaction involving a luxury apartment in Kiev that was tokenised into NFTs^5^. This transaction highlights the disruptive potential of NFTs in real estate transactions. In particular, these transactions may improving market liquidity and accessibility^6^. Furthermore, the launch of the cryprographic tokens “ERC404” on the Ethereum blockchain tokens is a prime example of the blurring lines between regular cryptocurrencies and Non-Fungible Tokens (NFTs). ERC404 tokens are a hybrid that combines the uniqueness and ownership characteristics of NFTs with the liquidity and tradability of cryptocurrencies like Bitcoin. This novel method not only challenges traditional categorisation within the digital asset field, but it also presents complex VAT/GST compliance issues^7^. As a result, these examples show the complicated and multidimensional nature of digital asset transactions, setting a larger conversation about the necessity for efficient VAT/GST compliance measures in the EU’s digital economy^8^.

The European Union (EU), with its different member states, has been at the forefront of addressing these difficulties, working towards a unified VAT/GST compliance policy with a specialized “VAT Expert Group” that advises the European Commission^9^. The *Skatteverket v. David Hedqvist *case (C-264/14)^10^, which established a precedent for the VAT treatment of Bitcoin transactions, had a significant impact on the broader world of digital assets inside the EU’s VAT Directive: The EU VAT Directive sets a harmonised framework for VAT taxation across member states, with room for national variations in implementation. The standard VAT rate in the EU cannot be less than 15%, with the potential for reduced rates of at least 5% on specified goods and services and exceptional rates based on prior agreements^11^.

*Skatteverket*^12^ emphasised the complexities of digital asset transactions and their implications for VAT compliance, raising concerns about the effectiveness of existing tax legislation and enforcement techniques. The verdict has implications beyond cryptocurrencies, including the fast-expanding market of NFTs since 2021, which represent unique digital ownership rights and have become a significant economic and cultural phenomenon^13^.

Despite the *Skatteverket*^14^ case’s importance, implementing its principles across EU member states has highlighted variations and obstacles in VAT/GST compliance methods for digital assets and NFTs that are in a fragmented state on how they are “created, listed, transacted, reported and taxed”^15^. This variation in implementation has highlighted the need for a more coordinated strategy to ensure fairness, legal certainty, and effective tax administration throughout the EU^16^.

Given this background, the aim of this research is to critically assess how the Skatteverket verdict affects VAT/GST compliance methods for digital assets and NFTs in the EU, as well as to investigate the consequences of harmonising VAT enforcement across member states. This article seeks to fill a gap in the present understanding of VAT compliance for digital assets by addressing the topic of how EU member states can manage the difficulties created by the digital gig economy within the existing VAT system.

This article aims to provide insights into the changing landscape of digital asset taxation by undertaking a comparative legal analysis of the *Skatteverket *case and following VAT compliance efforts across the EU. The study focuses on the relationship between legal precedents, *Skatteverket*’s relationship with national VAT implementations, and the distinguishing features of digital assets and NFTs. The research’s approach attempts to provide recommendations for developing a more consistent and effective VAT/GST compliance strategy in the EU’s digital economy.

In summary, this article is motivated by a desire to better understand the Skatteverket case’s influence on VAT/GST compliance for digital assets in the EU, as well as to find avenues towards harmonised tax enforcement across member states. The final aim is to help build a consistent, fair, and efficient VAT structure that reflects the dynamic character of the digital economy within the EU and its trade with the US and China and to help understand how ECJ classifications of digital platforms affect regulatory obligations and compliance strategies in the digital market^17^.

## Materials and methods

This study was founded on a comparative legal analysis, meticulously investigating the VAT/GST compliance regimes within the European Union, with an emphasis on digital assets, using the perspective of the *Skatteverket v. David Hedqvist*^18^ case. The methodology focused on analysing primary legal sources such as EU directives, member state legislation, and landmark case law.

### Case selection and methodology

The cases for the study were selected using a criteria-based methodology, with the goal of including rulings that have significantly changed the landscape of VAT/GST compliance for digital assets and services in the EU. The criteria for case selection were as follows.

1. **Relevance to the Digital Economy:** Cases were chosen because they have direct or indirect consequences for the taxation of digital services and goods/assets, such as cryptocurrencies and NFTs. The *Skatteverket*^19^ case is pertinent to the internet economy because it highlights the challenges that digital corporations, such as Amazon, encounter while implementing the EU’s VAT system. Amazon’s adjustments to comply with different VAT legislation across member states highlight the importance of a cohesive digital single market and harmonised VAT administration^20^.
2. **Jurisprudential Impact:** Priority was given to instances that contributed to the formation of legal principles or were essential in the interpretation of VAT/GST rules in the context of the EU’s digital economy.
3. **Citation & Precedent:** Cases that have been repeatedly cited in later legal decisions, research articles, and reports, demonstrating their importance and impact in developing VAT/GST compliance and enforcement techniques.

**Fig. 1:**
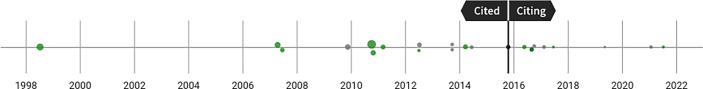
Precedent Map of the Skatteverket Case: Visualisation of Citation History for the Skatteverket Case

The precedent map visualises the citation history of the *Skatteverket*^21^ case over time (Fig. 1).

- Chronology: The horizontal line indicates a chronology that runs from around 1998 to 2022.
- Citation Indicators: The green dots throughout the timeline indicate when the Skatteverket case has been mentioned by other cases (mentioned) or when it has cited other cases (Citing).
- Cited (Left Side): The dots on the left side indicate when the Skatteverket case was mentioned in later court rulings. The grouping of dots around specific years may indicate periods of increased influence or relevance.
- Citation (Right Side): The dots on the right side represent the instances that the Skatteverket case has quoted. This depicts the legal precedents deemed significant to the *Skatteverket *case.
- Size of Dots: If the size of the dots coincides with the frequency or number of citations, larger dots indicate a case that has been mentioned more frequently, indicating a higher level of impact.

Therefore, Citation and precedent assist this text in understanding the evolution and application of law.

4. **Variability in Jurisdiction:** Cases from several EU member states were also considered in order to capture the variability in the application, including an interpretation of VAT/GST regulations across jurisdictions. In combination with the precedent analysis, this analysis helps to find examples of when VAT/GST laws have changed. This helps judges and legal professionals understand the legal logic behind VAT/GST decisions.

The comparative analysis employed in this article includes a thorough examination of selected examples pertaining to the overall EU VAT Directive and its implementation by member states. This investigation sought to find the legal principles established by the *Skatteverket *decision, as well as their application or evolution in subsequent jurisprudence involving digital assets. The analysis was supplemented with secondary sources such as scholarly discourse, European Commission papers, and European Court of Justice rulings to provide a thorough understanding of the legal complexities and trajectory of VAT/GST compliance regimes in the digital age.

This methodological approach enables a thorough examination of the VAT/GST compliance processes inside the EU’s digital economy, exposing the subtleties of legal precedent and its repercussions. Using a comprehensive and criteria-based case selection procedure, the research presents a cogent examination of the issues and tactics surrounding VAT compliance, laying the groundwork for future research and policy concerns in the field of digital taxes.

### Background of the Case C-264/14 (Skatteverket v David Hedqist)

*Skatteverket *ECJ Case C-264/14, or Skatteverket v David Hedqvist, was a highly relevant landmark case in EU VAT regulation of virtual currency transactions because, ultimately, “tax rules determine who should contribute to society”^22^. The ECJ Case C-264/14 (hereinafter referred to as the *Skatteverket *case) at hand concerns a preliminary ruling by the European Court of Justice (ECJ) on the application of Value Added Tax (VAT) to transactions involving the exchange of traditional “fiat” currencies for units of the virtual currency “Bitcoin” and vice versa^23^. The question was whether commercial Bitcoin exchange services were subject to VAT under the European Union’s VAT Directive (Directive 2006/112/EC)^24^.

The request for this preliminary ruling stemmed from a dispute between the Swedish Tax Authority (aldo called Skatteverket) and the Swedish citizen Mr David Hedqvist. David Hedqist intended to provide such exchange services through a company^25^.

Mr Hedqvist had intended to provide exchange services for both selling and buying Bitcoin for fiat currencies (such as the Swedish krona). The business model consisted in charging a margin on each transaction. These transactions raised the question as to whether the acquisition of Bitcoin could be classified as a provision of services in exchange for payment, and if so, whether they were eligible for exemption from VAT according to the regulations outlined in the EU VAT Directive.

The ECJ found that when a company charges a margin for transactions involving the exchange of traditional currency for Bitcoin (and vice versa), it constitutes a supply of services for consideration under Article 2(1)(c) of the VAT Directive. The reason for this is the direct connection between the service offered (currency exchange) and the payment received (margin). The Court then examined if converting fiat currency to Bitcoin should be exempt from VAT under Articles 135(1)(d)–(f) of the VAT Directive^26^. It specifically considered the following:

“Article 135(1)(d) exempts transactions involving deposits, payments, transfers, debts, checks, and other negotiable instruments”^27^^.^ The Court determined that Bitcoin transactions do not fall into these categories because they involve a direct method of payment between the parties.

Article 135(1)(e) exempts transactions involving currency, banknotes, and coins used as legal tender. The Court interpreted this provision broadly, ruling that it also applies to Bitcoin transactions because the parties accept it as a form of payment, just like traditional currencies, in line with working paper 049 of the European Commission on VAT Treatment of Bitcoin^28^. Thus, traditional currency exchanges for Bitcoin and vice versa are exempt from VAT^29^.

Article 135(1)(f), which applies to transactions involving shares, debentures, and other securities, was deemed inapplicable to Bitcoin exchanges because Bitcoin does not confer a property right or is not a security of comparable nature^30^.

The landmark *Skatteverket*^31^ case, ECJ Case C-264/14, signifies a historic turning point in the legal treatment of virtual currencies^32^. The decision establishes that, under the EU’s VAT Directive, Bitcoin exchange services for traditional currencies are not subject to VAT, bringing some legal clarification to EU jurisdiction^33^. This was an important milestone that acknowledged the distinct nature of Bitcoin transactions as services given for consideration, exempt under Article 135(1)(e), rather than the exchange of tangible objects.

This exemption from VAT for Bitcoin exchange services can be seen as a recognition of cryptocurrencies’ functional equivalency to traditional legal tender in certain transactional circumstances^34^. It reflects growing legal knowledge and response to the transactional realities in the Digital Single Market in the European Union which resulted from technological breakthroughs in digital currencies^35^.

### Case C-264/14 (Skatteverket v David Hedqist)’s formative impact and reasoning

In the *Skatteverket*^36^ case, the ECJ provided legal clarification by applying existing VAT rules to virtual currency. The Court decided that the exchange of Bitcoin for traditional currencies is a service rather than a supply of goods, which is crucially linked with the directive’s provision exempting transactions involving cash, bank notes, and coins used as legal tender from VAT. The ECJ’s reasoning suggested that Bitcoin functions as a payment method and should be regarded similarly to other legal tenders under the VAT system, thus following the stance of the Value Added Tax Committee in Working Paper No 811^37^. Aparicio’s (2015) research is notable for its early identification of digital currencies in European VAT law, demonstrating the ECJ’s ability to navigate the complicated application of traditional tax laws to digital currencies. Aparicio’s work highlights the ECJ’s pioneering work in integrating digital currencies into legal frameworks, providing a precedent for future legal arguments^38^. Building on this, Veerpalu’s Baltic Journal of Law & Politics paper examines how technological decentralisation affects VAT rules. Veerpalu examines the ECJ’s decision-making process to highlight the Court’s involvement with Bitcoin’s decentralised character, which works outside the traditional financial system^39^. In 2022, Lee and Van de Looverbosch examined the link between property rights and data in the financial sector, moving from VAT to data governance. Their research shows cryptocurrency’s legal and regulatory challenges. Lee and Van de Looverbosch (2022) improve our grasp of cryptocurrency law by applying data governance ideas to the financial industry and addressing ownership, privacy, and regulation^40^. Pistone’s 2008 examination of the ECJ’s direct taxation case law provides a crucial context for understanding the Court’s taxation approach before cryptocurrencies’ widespread acceptance. This historical perspective helps us understand the legal grounds that would subsequently affect the ECJ’s digital currency and VAT decisions and its developing taxation position in the digital era.

*Skatteverket*^41^ also demonstrates that the application of VAT exclusions to Bitcoin transactions is consistent with the idea of fiscal neutrality^42^. By classifying Bitcoin as a means of payment rather than a commodity or security, the ECJ insured that Bitcoin transactions would not be disadvantaged by VAT when compared to regular currency transactions, which are often VAT-free^43^.

This ECJ ruling reflects a broader trend in the digital economy, in which legal definitions and frameworks are gradually responding to the realities of digital currencies and assets. The ECJ’s contrast between Bitcoin and other financial products, such as securities or shares, which are not excluded under Article 135(1)(f), highlights cryptocurrencies’ unique status in the financial landscape.

### Case C-264/14 (Skatteverket v David Hedqist)’s application of precedent and reasoning

The European Court of Justice (ECJ) cited judgements that, while not directly dealing with virtual currencies and NFTs, established rules applicable to the regulation of Bitcoin transactions for VAT purposes^44^. These principles centre around the nature of the supply (goods vs. services), the use of VAT exemptions for financial services, and how these exemptions are interpreted considering the EU VAT Directive’s objectives^45^:

Distinguishing *Granton Advertising BV*^46^, the ECJ drew a line between digital services and goods, emphasizing the type of the supplier in establishing VAT eligibility. *Granton Advertising BV *focused on whether the sale of discount cards qualified as a transaction involving “other securities” or “negotiable instruments” for VAT exemption reasons. It was determined that it is not exempt under these categories. In *Skatteverket, *the ECJ explained that Bitcoin transactions do not entail the delivery of tangible goods but rather the provision of services, notably financial services, which may be exempt under Article 135(1)(e) of the VAT Directive.

Similarly, citing *Le Rayon d’Or (C-151/13) and Consideration of Services*^47^, the ECJ considered the question of consideration for VAT: Le Rayon d’Or addressed whether payments paid for healthcare services provided to residents of care homes constituted consideration for VAT purposes, and concluded that they did. In *Skatteverket*, the consideration was the margin contained in the exchange rate for Bitcoin transactions, reinforcing the premise that the presence of consideration is required for a transaction to be VAT-chargeable. However, despite the presence of consideration, Bitcoin transactions were exempt because they were classified as financial services.

The ECJ’s argumentation line paralleled the argument in *The First National Bank of Chicago (C172/96) and Financial Transactions*^48^: The *First National Bank of Chicago *addressed the VAT status of foreign exchange transactions, emphasising that profits made from currency spreads constituted consideration for VAT purposes but were exempt as financial transactions. Similarly, *Skatteverket *treated Bitcoin exchanges as VAT-exempt financial transactions. The case demonstrates the ECJ’s continuous stance to recognise currency exchanges, whether virtual or traditional, as VAT-exempt financial services.

The ECJ’s reasoning in *Skatteverket v Hedqvist *for excluding Bitcoin transactions from VAT is based on a uniform application of VAT Directive principles across different types of transactions. Comparing and contrasting these situations reveals several significant rationales:

1. **Nature of the Supply**: The ECJ distinguishes between the supply of products and the supply of services, with Bitcoin transactions falling under the latter. This classification is critical for determining the appropriate VAT exemptions.
2. **Consideration and Financial Services**: The presence of consideration is required for VAT to apply; nevertheless, transactions classed as financial services that meet certain conditions (such as currency exchanges) may be exempt from VAT.
3. **Objective of VAT Exemptions**: The exemptions are intended to alleviate administrative difficulties and complexity in taxing financial transactions. By exempting Bitcoin exchanges, the ECJ supports the goal of simplifying VAT treatment for financial services.

In short, the *Skatteverket *ruling extends traditional VAT rules to the unique environment of virtual currency^49^. The comparison with the referenced judgements demonstrates the ECJ’s respect to these principles while also acknowledging the distinctive nature of Bitcoin transactions as financial services, which exclude them from VAT. This strategy ensures a coherent and consistent VAT system that adapts to the changing nature of financial transactions in the digital platform economy.

### The impact of the technical aspects of digital assets on Case C-264/14 (Skatteverket v David Hedqist)

On 22nd October 2015, when the *Skatteverket *ruling was announced, blockchain technology was still in its early stages of development and NFTs did not exist. This indicates that the decision was made with outdated technology in mind without considering recent advancements^50^. Consequently, digital transactions and the legal structures designed to regulate them entered a period of significant disruption. During this significant era, Bitcoin emerged since its invention in 2008 as a pioneering cryptocurrency, instigating a flurry of technological and legal progressions within the digital economy to address the shortcomings of the traditional currency system^51^. The ascent of Bitcoin to prominence can be attributed to its decentralised structure, an innovative notion enabled by blockchain technology^52^. Blockchain facilitated peer-to-peer transactions and obviated conventional financial intermediaries like banks.

Musleh et al. (2019) said that these decentralised peer-to-peer Blockchain transactions significantly improved the level of security and transparency in financial markets’ transactions. A dispersed network of participants had access to a synchronised, immutable ledger that enabled this to be achieved. By eliminating any potential point of failure, this ledger significantly enhanced user autonomy and marked a substantial departure from preceding financial systems^53^.

Nevertheless, the progression of digital assets continued with the advent of Non-Fungible Tokens (NFTs), which, despite operating on the same blockchain infrastructure as Bitcoin, were structurally and functionally distinct. NFTs are characterised by their exclusivity and indivisibility, which serve as indicators of ownership for digital products and allow particular usage in smart contracts^54^. Sakız and Gencer (2021) assert that NFTs lack the facility of transferability akin to Bitcoin and are incapable of one-to-one exchange. Legal challenges have been notably complicated by this distinctive characteristic of NFTs, especially in comparison to the more standardised iteration of Bitcoin. The legal implications of transferring intellectual property rights, proof of ownership, and rights via smart contracts have all posed significant challenges that have been largely disregarded by current legal frameworks^55^.

The *Skatteverket *case exemplifies the ongoing challenge faced by the judicial system in staying abreast of these swift technological advancements. As a result of the introduction of digital assets like Bitcoin and, subsequently, NFTs, global regulatory systems have been compelled to be reevaluated and modified. Legislators and legal specialists are perpetually striving to implement interim rules and refine legal terminology to reconcile the evolving technological landscape with well-established legal doctrines. This undertaking has often led to ambiguous legal interpretations and provisional legislation, illustrating the legal system’s limited capacity to fully comprehend and integrate the complexities introduced by emerging digital advancements.

The *Skatteverket *case highlights the legal system’s persistent struggle to accommodate rapid technological advances. Global regulators and legal systems are always working to update and clarify the legal frameworks that govern digital assets like Bitcoin and NFTs. This frequently leads to ambiguous legal language and interim rules intended to bridge the gap between developing technologies and existing legal doctrine. When the CJEU considered *Skatteverket*, both Advocate General Kokott and the court determined that the transactions in question were exempt under Article 135(1)(e) because they involved ‘transactions... concerning currency, bank notes, and coins used as legal tender’. The ambiguity arose over whether ‘legal tender’ was intended to qualify as ‘currency’, raising the question of whether the exemption could apply to non-regulated currencies. This was complicated by the Directive’s language variations; for example, the German text implied an exemption for ‘currencies... which are legal tender’, whereas the Finnish text suggested it only applied to banknotes and coins. The court defined ‘currency’ as a type of payment that, if accepted by transaction parties as an alternative to legal tender and serves no other purpose, constitutes a financial transaction. Following the principle of fiscal neutrality, the advocate general agreed, arguing that items used solely as payment methods, such as Bitcoin, should be treated as legal tender, distinguishing them from goods like gold or cigarettes, which have other uses besides payment^56^.

Existing legal doctrine is designed to uphold the principle of neutrality in the VAT system, despite being aware of inherent economic inefficiencies. It acknowledges the VAT’s biases and distortions yet continues to strive for an equitable solution. The doctrine does not ignore its shortcomings; rather, it actively seeks to address and mitigate them while maintaining the integrity and functionality of the tax system^57^.

## Results: analysis of VAT/GST in the digital economy

The *Skatteverket *ECJ ruling marked a significant change in the Value Added Tax (VAT) enforcement landscape within the European Union. This part looks at the main laws and rules that control transactions in the digital economy, along with the current VAT rules for digital services^58^.

Value-added Tax (VAT), also known as goods and Services Tax (GST) in some regions, is a comprehensive tax levied on products, services, and intangibles consumed primarily by individuals. VAT/GST is used by around 168 countries as a substantial income source, with the United States being a notable exception. This tax system applies to purchases made for personal consumption rather than business purposes, with enterprises in the supply chain functioning as tax collectors on behalf of the authorities.

VAT/GST is introduced in stages, with each business in the supply chain charging and remitting VAT based on its profit margin, ensuring that the tax is gradually collected at each transaction point. VAT/GST is ultimately paid for by the final consumer, even though it is collected at every point of the supply chain, including corporate transactions. The tax is often levied at a regular rate, like a sales tax.

The place of supply—with differing rules for business-to-business (B2B) and business-to-consumer (B2C) transactions—and the time of supply are important factors in determining VAT/GST duty. For B2B services, the place of supply is often where the client is located, whereas, for B2C transactions, it is where the supplier is situated, with some exceptions for certain types of services. The time of supply, or tax point, determines when the tax is payable, allowing for accurate collection and reimbursement. Some countries have examined or adopted VAT exclusions for cryptographic digital assets, reflecting the changing nature of taxable products and services^59^.

### EU’s VAT framework for digital services

In the European Union, VAT (also called Goods and Services Tax—GST) is crucial for the single market’s tax system. The EU’s VAT system focuses on the place of supply rules, which are designed to allocate tax revenue among member states based on where consumers are located within the EU. This approach aids in preventing tax evasion and ensures that VAT is accurately collected on cross-border transactions within the EU^60^. The EU VAT system follows the VAT Directive, which is also called Directive 2006/112/EC and is designed to tax private consumption indirectly by applying VAT to all stages of the production and distribution process, not just the final sale to private consumers. This all-stage turnover tax ensures that the burden of VAT ultimately falls on the consumer despite being collected in fractional payments throughout the supply chain. The aim of this approach is to avoid the risks of incorrect VAT filing or tax fraud, as these fractional payments spread the risks across multiple transactions and the risk is not concentrated at the point of final sale.

This is achieved by making the taxable person such as a business supplying goods or services the VAT payer who is required to collect the tax from the customer and to send it to the national tax authorities. At the same time, the taxable person’s right to deduct the VAT paid creates “VAT neutrality”. Consequently, a business is only called upon to pay VAT to the added value, and thus the tax does not cascade throughout the supply chain.

Applying these principles to digital assets, if exchanges of money or currency for a specific digital asset were treated as taxable supplies of services, the VAT system would function similarly. For example, if a digital asset exchange platform (taxable person A) charges a fee for converting fiat currency to a digital asset, VAT would be applied to this service fee. If this platform then uses services from another business (taxable person B), it can deduct the VAT paid on these services against the VAT collected from its customers, ensuring that only the added value is taxed. This is a challenge when a service is rendered virtually in the metaverse, for instance, in the platform “Second Life”, as the purchase of virtual land via a currency yields different results when assessed by local and federal courts in Germany^61^.

In essence, even in the context of digital currencies, the aim is to tax final consumption by private individuals. Since digital currency exchanges provide a service to consumers, the VAT applied reflects the consumption of this service. The consumer ultimately bears the VAT cost, aligning with the system’s goal to tax private consumption without burdening businesses with the tax, provided those businesses are part of the VAT system and make taxable supplies^62^.

VAT application complexities changed and grew across European Union (EU) Member States after the *Skatteverket *case. These complexities are embedded within the broader legal and regulatory framework governing the Economic and Monetary Union (EMU) and include the taxation of different types of cryptographic digital assets.

Article 3 1 lit c of the TFEU gives the EU exclusive monetary policy authority over Euro Member States. This emphasises the EU’s centralised management of the single currency and monetary policy, preventing Member States from issuing their own currency or adopting monetary policies that conflict with the Euro. The European Central Bank and national central banks can issue euro banknotes under Article 128(1) of the TFEU. The Euro is the only currency in the Eurozone with legal tender status. Paragraph 3 of the same article authorises coin issuance to Member States within ECB-approved volume limits. Article 10 S2 of the Euro Introduction Directive introduces the Euro as the EU’s single currency^63^. This means that while the Euro is the mandated single currency for Eurozone countries under the TFEU, Bitcoin is not recognised as an official currency within the EU^64^. Yet Bitcoin and other cryptocurrencies are not explicitly prohibited under the TFEU. These allow consenting parties to trade goods and services. However, these currencies are not legal tender like the Euro. Municipalities and state organs may accept other forms of value exchange under certain conditions, but EU, national, and local laws can intersect in complex ways, especially with digital assets and cryptocurrencies.

Understanding these legal nuances is crucial when considering cryptocurrency transactions’ VAT tax implications.

### Disparities in VAT/GST implementation across the EU

The *Skatteverket *ruling has thrown some light on VAT exemptions, yet internet businesses continue to face a complex and uneven VAT enforcement landscape throughout the European Union. This disparity in VAT application and interpretation among EU countries presents barriers to cross-border trade, hindering efforts to standardise VAT processes and fully realise a digital single market^65^. To address these problems, EU Member States must strengthen collaboration and alignment to ensure effective VAT management for the increasing digital transactions across the union^66^.

Several ECJ classification incidents demonstrate the disparities in VAT schemes and their application:

*Commission v. Luxembourg *(C-502/13): This case criticises Luxembourg’s reduced VAT rates on digital books, claiming they violate the EU VAT Directive. It contrasts with the *Skatteverket *decision, which dealt with VAT exemptions for Bitcoin, by highlighting the VAT treatment split between digital and physical commodities, as well as the issues of digital product taxation.

*Commission vs France *(C-479/13): Like the Luxembourg case, this dispute surrounds France’s application of reduced VAT rates on digital books, which is seen to violate the EU VAT Directive. Unlike the Bitcoin exemption case, it digs into the VAT problems of the digital single market, highlighting the EU’s struggle to harmonise VAT on digital and physical commodities.

*European Commission v. Germany *(C-616/15 P): This lawsuit focuses on a specific VAT plan for travel brokers, which contrasts with the Bitcoin VAT exemption. It focuses on broader VAT compliance difficulties across several areas of the EU’s internal market, demonstrating the varying implementation of VAT legislation.

*FRANCK d.d. Zagreb in Ministarstvo financija Republike Hrvatske *(C-801/19): The focus here is on VAT exemptions for specific financial transactions, which differs from the Bitcoin exemption issue. It investigates complex transactions such as bills of exchange to demonstrate the EU’s diverse interpretations of VAT exemptions across economic activities.

*Vega International Car Transport and Logistics—Trading GmbH v. Dyrektor Izby Skarbowej w Warszawie *(C-235/18) examines the complexities of VAT exemptions for fuel cards used in transportation services. This decision is significant because it distinguishes these provisions from other financial transactions involving cryptocurrencies such as Bitcoin. The European Court of Justice (ECJ) clarified the application of VAT Directive articles 168 and 173(2)(c), stating that overhead costs transferred to the customer via interest on the finance portion of a hire purchase are still considered part of the goods’ supply price. Crucially, the Court stated that Member States may not use an apportionment method that disregards the initial value of the goods at supply. Such a method would not ensure a precise allocation when compared to the standard turnover-based key, affecting input tax deductions^67^.

*In Dietrich v. Hessischer Rundfunk, C‑422/19*^68^*, *the European Court of Justice (ECJ) addressed the legal tender status of euro banknotes and the acceptability of cash payments. Diverging from the Bitcoin VAT exemption highlighted in *Skatteverket v Hedqvist*, the case exposed broader issues about money and payment systems in the EU. The German court sought ECJ guidance on whether the refusal by Hessischer Rundfunk to accept cash for television license fees violated EU principles regarding the euro as legal tender. The ECJ determined that while Member States could mandate public administrations to accept cash, they could also impose limitations based on public interest, provided these are proportionate. This ruling reflects the balance between recognizing the euro’s legal tender status and allowing Member States some autonomy in managing their public administration’s payment procedures^69^.

In *Bookit Ltd v Commissioners for Her Majesty’s Revenue and Customs *(Case C-607/14), the European Court of Justice (ECJ) considered the VAT exemption status of “card handling” services used to purchase cinema tickets online or over the phone. Unlike the *Skatteverket *decision, which exempted Bitcoin transactions from VAT, the ECJ determined that card processing services do not qualify for the exclusions offered for payments and transfers under Article 135(1)(d) of the VAT Directive and if a person is solely “responsible for the transmission of data associated with a transfer, rather than the transfer itself”. This ruling highlights the court’s complex approach to establishing the scope of VAT exemptions, emphasising that not all services that facilitate payments qualify for exemption^70^.

The European Court of Justice (ECJ) clarified in the *Revenue and Customs Commissioners v British Film Institute *(C-592/15) case that the VAT exemption for cultural services under Directive 77/388 Article 13A(1)(*n*) lacks specificity and thus cannot be invoked directly before national courts without domestic transposition. This contrasts with *Skatteverket*, where the ECJ determined Bitcoin transactions exempt from VAT, immediately affecting VAT compliance procedures without any ambiguity in interpretation or implementation^71^.

### Impact of Case C-264/14 (Skatteverket v David Hedqist) on subsequent cases and compliance challenges

The key verdict in *Skatteverket *has had a dramatic impact on VAT compliance across the European Union, notably in terms of digital transactions and virtual currencies. A review of the cases in which *Skatteverket *is cited sheds light on how this legal concept has influenced VAT compliance procedures in a variety of industries, including financial services and the digital economy’s trade of goods and services.

Notable is the case of *Coinstar Ltd v Revenue and Customs Commissioners *(2016) for its alignment with and expansion of the *Skatteverket *decision, and it highlights the greater applicability of VAT exemption principles to innovative financial services. At this landmark case, the First-tier Tribunal (Tax Chamber) examined Coinstar’s coin-to-voucher exchange terminals, which enabled a practical exchange service by providing vouchers redeemable for cash at supermarkets. The tribunal recognised Coinstar’s service as a VAT-exempt financial transaction, following the precedent established by the European Court of Justice in *Skatteverket*^72^. This decision emphasises the adaptability of VAT exemptions, expanding their scope beyond digital currencies to include physical currency exchange services, so establishing an important precedent for the regulation of similar financial services under VAT law^73^.

The ECJ referral in the *X‑Network*^74^ case is a stark contrast to *Skatteverket*, which pushed VAT law into subtle disputes over voucher classification and supply chain complexity. *Skatteverket *set a precedent for VAT-exempt digital transactions by classifying virtual currencies as financial services. In contrast, the *X‑Network *recommendation delves further, examining the VAT implications for single- and multi-purpose vouchers, particularly in multi-layered transactions. It asks whether a voucher’s multi-stage transfer across multiple jurisdictions triggers VAT duty at each transaction point, potentially resulting in double taxation. This legal challenge focuses on determining when VAT applies, with the goal of reconciling the VAT Directive’s position on single-purpose vouchers with the realities of digital commerce’s complex supply chains^75^.

*Skatteverket *impacted a subsequent case, *Target Group Ltd. v. Revenue and Customs Commissioners*. This discussion focused on the VAT treatment of loan administration services given to a lender by a business. The question was whether these services met the conditions for a financial transaction as specified in Directive 2006/112 article 135(1)(d) and thus qualified for VAT exemption.

If performed by a third-party loan servicer subsequent to the loan’s authorization, the Court of Appeal determined that such services are not exempt from value-added tax (VAT). As transactions involving payments, debts, or bank account operations, Target Group’s principal services to Shawbrook Bank, including loan account management and payment processing for borrowers, were determined to be taxable and subject to VAT. Informing its decision was an extensive corpus of case law from both the United Kingdom and the European Union, which included precedents from the most recent CJEU rulings. After the United Kingdom left the jurisdiction of the European Union, these decisions were enshrined in British legislation. For this purpose or reason, the European Union (Withdrawal) Act 2018 was passed^76^.

Subsequently, this decision was upheld by the Supreme Court, which underscored the necessity of personal involvement in the execution of the transfer or payment for the payment VAT exemption to be applicable. Directives to a third party merely do not suffice to initiate a transfer or payment. By mandating “functional participation and performance” in its implementation, the Supreme Court upheld a strict interpretation of the payment VAT exemption^77^.

To ensure compliance with the stipulations outlined in the Supreme Court’s decision, organisations operating in the payment and gig sector ought to evaluate their protocols^78^. Hence, their VAT positions, and in some cases, whether their services are eligible for payment VAT exemption, may need to be reconsidered following this decision. Moreover, the judgment affects the VAT classification of the services and the hence the service portfolio of Payment Service Providers, e‑money and cryptocurrency providers, among others^79^.

In the case of *TMD Gesellschaft für transfusionsmedizinische Dienste mbH v Finanzamt Kassel II—Hofgeismar *(C-412/15), the European Court of Justice (ECJ) clarified that the supply of plasma, a component of human blood used in the manufacture of medicinal products, is exempt from VAT under Article 132(1)(d) of Directive 2006/112/EC. This decision reflects the ECJ’s broader view of VAT exemptions in healthcare, comparable to the *Skatteverket *case, which found that transactions involving virtual currency (Bitcoin exchanges) are exempt from VAT under the same directive. While the TMD decision broadens VAT exemptions to biological materials used for therapeutic reasons, in contrast to *Skatteverket’*s emphasis on digital currency transactions, both rulings have the goal of easing access to vital services and goods by exempting them from VAT. This convergence illustrates the ECJ’s commitment to interpret VAT law in a way that benefits the public interest in both the healthcare and financial markets.

*Skatteverket *has had a considerable impact on VAT compliance procedures, as indicated by how the decision has been used to interpret the VAT treatment of a wide range of financial transactions. The verdict’s explanation of what qualifies as a VAT-exempt financial service has made VAT legislation across the EU more consistent and predictable, which implies benefits for both businesses and tax authorities^80^.

Furthermore, the *Skatteverket *case has implications for the regulatory framework governing developing technologies and digital markets. The decision establishes a precedent for deciding the VAT treatment of emerging financial technology and services, including those that do not include cryptocurrencies, as the incidence of digital transactions grows. This emphasises the importance of keeping value-added tax (VAT) legislation up to date with technological improvements, thereby protecting against compliance requirements that stifle innovation or place unnecessary restrictions on digital firms.

*Skatteverket *adds to the larger discussion on the importance of integrating the VAT framework inside the European Union’s digital single market beyond the immediate implications for financial transaction compliance. Given the eroding differentiation between traditional financial services and emerging digital platforms as a result of digital currencies and other fintech advancements, establishing a transparent and consistent value-added tax (VAT) framework becomes increasingly important in promoting international trade and investment in digital industries.

The *Skatteverket *decision, as well as later applications in *Coinstar Ltd and Target Group Ltd*, demonstrate the complex interplay that exists between technology improvements, economic progress, and VAT regulation. By clarifying the VAT classification of financial and digital services, these verdicts provide a more hospitable climate for digital commerce, fostering expansion and advancement in the European Union’s digital economy.

To summarise, *Skatteverket *has had a broad and complex impact on EU VAT compliance procedures. The reference to the verdict in future instances transformed the VAT environment for a wide range of financial services and transactions, advancing the values of transparency, uniformity, and equality in VAT legislation. *Skatteverket’s *guiding principles will surely remain the foundation for assessing VAT conformity as the digital economy evolves. This guarantees that tax policy keeps pace with technological changes and supports the smooth integration of emerging technology into the European market.

### Complexity of NFT VAT treatment

The verdict from *Skatteverket *provided clarified the VAT Treatment of Bitcoin. The issue of NFTs remains ambigious^81^. The lack of a clear definition, combined with the unique properties of the tokens and the ability to integrate fungible and securities qualities, leaves the VAT application context unclear.

For example, Cryptocurrency NFT-hybrids, such as the ERC404 token on Ethereum is a step in the technical development of NFTs and cryptocurrencies. ERC404 can be imagined as having a one-of-a-kind trading card that is owned, and at the same time, pieces of it can be traded just like money. Combining NFTs and fungible cryptocurrencies thus blurs the distinction between property and currency. Unlike the unique non-fungible NFTs that are captured by standard ERC721, ERC404 generates a hybrid with the liquidity and trading flexibility of fungible tokens. ERC404 tokens are hybrid in nature, combining the features of ERC20 (fungible tokens similar to cryptocurrencies) and ERC721. This means that each ERC404 token is tied to an NFT but has cryptocurrency liquidity, potentially revolutionising digital asset ownership and trading^82^.

As a result, case-specific analysis is required, as the EU VAT Committee articulates in Working Paper 1060.

Working Paper 1060 of the EU VAT Committee provides guidance on the VAT treatment of growing digital assets like cryptocurrencies and NFTs^83^.

The EU VAT Committee’s working paper on non-fungible tokens (NFTs) examines the VAT implications of NFT-related transactions, drawing on insights from various EU member states, including Spain, Belgium, and Norway. This analysis highlights the various approaches to VAT application on NFTs in the European Union.

Spain considers certain NFT transactions, notably those that provide buyers the right to use digitally modified images, to be electronically supplied services (ESS). This classification by the Spanish General Directorate of Taxes closely ties NFTs with digital content distribution, making them subject to VAT as services.

In response to a parliamentary query, Belgium’s Ministry of Finance advises that NFT transactions are VAT chargeable. NFTs are regarded as “a digital collection” or “an object of digital art”, implying a broader understanding of NFTs that includes their role as collections or art rather than focusing exclusively on their utility or the rights they grant. Belgium’s inclusion of NFTs within the scope of VAT-chargeable transactions underscores the expanding fiscal acknowledgement of digital assets and aligns with the European Court of Justice’s approach of broadening the interpretation of financial instruments and currencies in the context of VAT directives^84^.

Norway, which is not a member of the EU but is part of the European Economic Area, considers transactions involving NFT artworks to be ESS, with minting services being outside the purview of VAT. This position recognises the digital nature of NFT artworks and distinguishes them from the minting process itself.

These country-specific insights demonstrate a shared definition of NFTs as services, particularly within the ESS category, reflecting their digital essence and the rights or access they provide to digital assets. However, there are variations in how each country classifies the broader implications of NFT transactions, whether as art, a digital collection, or, more narrowly, as ESS, depending on the rights conferred.

The various approaches highlight the dynamic character of VAT policy in reaction to the digital economy’s new assets, such as NFTs. They also highlight the difficulties tax authorities confront when categorising emerging digital assets that do not fit neatly within established VAT systems. Companies and tax authorities struggle to ensure NFT marketplaces follow VAT regulations uniformly. The lack of a common framework to handle NFTs’ unique characteristics makes it less likely that enterprises would obey VAT regulations and harder to build a robust and logical EU NFT tax policy^85^. As the VAT Committee continues to investigate NFTs, these first observations from Spain, Belgium, and Norway provide a framework for future talks and perhaps harmonised VAT treatment across the EU^86^. This framework aims to level the playing field for businesses that work in the digital economy and make cross-border transactions easier by making sure that VAT responsibilities are consistent and clear across all Member States^87^.

Yet, VAT compliance remains a very cloudy legal landscape, especially when it comes to NFTs and Bitcoin: In the constantly changing world of digital assets, it is still not clear how the VAT rules should be put into place. If a business deals in digital assets like cryptocurrencies or NFTs, they have trouble keeping track of its VAT responsibilities because it does not know how to group the transactions. It is challenging for businesses in the digital economy to follow the rules because some digital services have unclear VAT treatment, and there is a chance of difference across member states, particularly due to different legal points of contact and various terms used^88^. Due to the legal uncertainty, it is more difficult to create a consistent and open method for VAT enforcement. The complicated VAT landscape in the digital age requires businesses to constantly adapt to new laws^89^.

The *Skatteverket case *makes clear the urgent need to set up a unified system for VAT enforcement right away that works with the complicated digital economy. Due to the fact that VAT is transposed differently in each EU member state, the VAT treatment of NFT transactions is complicated, and there is legal uncertainty surrounding VAT compliance in the area of digital assets. Therefore, reforms are needed right away^90^. Adopting a plan that makes sure VAT enforcement methods are the same across all countries could help lawmakers solve these problems and give businesses trust and openness during VAT law talks^91^. Policymakers can make sure that the EU’s digital economy is fair and equal by pushing people to work together and talk to each other. This will help businesses grow and meet VAT standards in a legal landscape that is constantly changing^92^.

## Discussion and results

### Interpretation of results

The results highlight a multifaceted and intricate obstacle that the European Union (EU) must surmount in order to standardise the enforcement of value-added tax (VAT) in the swiftly developing digital economy^93^. A momentous legal juncture was reached with the *Skatteverket *case, which was instrumental in redefining VAT compliance for cryptocurrencies and Non-Fungible Tokens (NFTs)^94^. These developments underscore the critical need for a legal structure that is sufficiently flexible and resilient to account for the distinct attributes of digital assets that have “posed numerous widely discussed challenges to public (administrative) law both within the EU regulatory environment and at a global level”^95^. Digital assets and, among them, currencies are not necessarily a “unitary category of assets” but a fluid series of types of assets with unique sui generis characteristics^96^.

A significant discrepancy has arisen regarding the interpretation and implementation of VAT legislation among EU Member States due to the *Skatteverket *ruling. The realisation of a cohesive digital single market is impeded by this fragmentation, underscoring the critical necessity for a standardised regulatory approach and a cohesive platform that does not disadvantage EU businesses when compared with their American and Chinese counterparts^97^. The treatment of NFTs, which are intricate digital assets that challenge traditional VAT frameworks, serves as a prime example of the necessity for legal clarity and consistency. In addition to hindering adherence to regulations, these discrepancies stifle innovation and hinder investor protection as companies contend with an unpredictably changing regulatory environment^98^.

The analysis it offers aligns with the perspectives presented by Chiara Zilioli in her article “Crypto-assets: legal characterisation and challenges under private law”. Zilioli’s (2020) article explores the wider ramifications that digital assets may have on established legal frameworks. Zilioli’s (2020) analysis of crypto-assets in the context of private law underscores the imperative for legal systems to adjust to digital advancements similarly. This alignment further supports the idea that the intricate nature of the digital economy necessitates nuanced legal reactions that protect the rights and responsibilities of citizens while not impeding technological advancement.

Zilioli (2020) draws a parallel between the judicial classification of crypto-assets and the rights bestowed upon them and our conclusions regarding the intricacies of VAT treatment^99^. This emphasises the critical nature of reaching an international consensus or implementing global standards to effectively tackle the crypto-asset phenomenon. Adopting an international strategy would effectively address the VAT compliance obstacles that arise from the digital economy, especially considering the worldwide scope of digital transactions.

Moreover, Zilioli’s (2020) investigation into enforcement concerns pertaining to crypto-assets highlights the complexities inherent in guaranteeing legal safeguards within decentralised networks. This further substantiates our discourse regarding the imperative for regulatory coherence across the European Union. The difficulties associated with regulating rights and responsibilities in decentralised crypto-asset networks are comparable to the intricacies we witnessed in VAT compliance; this underscores the need for a unified and cooperative regulatory approach^100^.

Given the aforementioned obstacles, this article proposes that a comprehensive strategy be employed to achieve consensus and clarity on VAT enforcement within the digital economy^101^. This requires the establishment of a standardised framework for value-added tax (VAT), periodic evaluations of legislation to align with technological progress, and improved cooperation across national borders. These measures would not only promote adherence but also cultivate a regulatory atmosphere that is supportive of novel ideas and approaches.

Moreover, the European Union’s endeavours to develop a Digital Taxation Directive and form a Digital Single Market Task Force are praiseworthy measures taken to tackle these obstacles^102^. These endeavours serve as prime examples of the creativity, cooperation, and adjustment required to address VAT compliance challenges in the digital age. Furthermore, the collaboration between tax authorities and technology companies such as Shopify and Microsoft to develop practical solutions for VAT compliance demonstrates the significance of stakeholder engagement^103^.

In summary, our research outcomes, when combined with observations, emphasise the critical need for legal and regulatory structures to adapt and develop alongside the digital economy. The *Skatteverket *case has brought to light the intricate nature of VAT enforcement and the wider obstacles that crypto-assets present, as well as the need for industry conversations^104^. As a result, a collaborative, inventive, and adaptable strategy is required. While tax authorities in Sweden have started collaborating with industry players to develop transparent and similar digital VAT regulations for Bitcoin and digital services in order to guarantee consistent processes throughout the nation, these efforts have not been fruitful yet^105^. Meanwhile, ongoing industry talks in Germany have made it possible for tax authorities to swiftly alter digital VAT laws to alleviate the negative impacts of the system on the growth of industries, highlighting the need of periodic adjustments to address shifting challenges in the dynamic context of the digital economy^106^. The EU can only hope to achieve a harmonised VAT compliance regime that accommodates the dynamic nature of the digital economy while ensuring fair and equitable taxation by implementing such a comprehensive strategy.

### Comparative analysis with the US and China

Addressing VAT neutrality in the context of cryptocurrency transactions necessitates a thorough examination of how this principle is interpreted and applied in various jurisdictions, particularly the approaches of the United States and China. The discussion about VAT neutrality—ensuring that VAT does not distort economic decisions and competition—is critical to understanding how different countries approach cryptocurrency taxation. This comparison will include key perspectives and regulatory stances from both countries, as well as the implications for businesses and consumers in the ever-changing digital financial landscape.

#### VAT neutrality

VAT neutrality is a basic principle applied in the VAT tax system to ensure that the levies charged under VAT do not unfavorably prejudice or put some businesses or business transactions at a competitive edge. VAT neutrality dictates that the tax under consideration should be neutral not to affect choices undertaken by businesses while passing through the chain and to the ultimate consumer. This principle is particularly hard to implement when considering the unique properties of digital assets.

#### The US: a focus on property and capital gains taxation

The US relied instead on state-level sales taxes as the major means of taxing consumption. Unlike VAT, which is collected at every stage of the manufacturing and distribution process, sales tax is normally levied solely at the point of sale to the final consumer. This distinction yields a less complex tax administration system but lacks the efficiency and revenue-generating capacity of a VAT system.

The United States does not have a federal VAT system due to historical reliance on other taxes at the federal level, unlike European countries that have embraced VAT as s significant revenue source. Introducing a different tax system like VAT would require significant modifications to current laws and may face opposition due to worries about government expansion^107^. The approach of the US to cryptocurrency taxation focus is on capital gains and property taxes. The Internal Revenue Service (IRS) has provided Notice 2014-21, 2014-16 I.R.B. 938 that treats cryptocurrencies as property for tax purposes. This means that capital gains tax will apply to transactions involving cryptocurrencies^108^. This approach aims to integrate cryptocurrency transactions into the existing US tax framework without needing a VAT system^109^.

#### China has centralised control and comprehensive regulations

China’s approach to cryptocurrencies and VAT neutrality can be gleaned from its overall regulatory environment and stance on digital assets. The Chinese government has imposed strict controls on cryptocurrency activities, including trading and mining bans, demonstrating a prioritisation of financial stability and control over the digital economy. Although China has a VAT system, the direct application of VAT neutrality principles to cryptocurrency transactions is complicated due to outright prohibitions on such activities^110^.

However, discussions about the taxation of digital goods and services, including the potential use of VAT, indicate that there is ongoing consideration of how to integrate digital assets into the existing tax framework while maintaining control and oversight. An explainer by the Shanghai Municipal Tax Service was published and then deleted. This document, initially interpreted by some as signalling a possible relaxation of China’s strict crypto ban, clarified that there is taxation on income generated from virtual currencies obtained and sold at a higher price. Despite speculative hopes for policy changes towards cryptocurrency acceptance, mainland legal experts assert that the explainer does not indicate a shift in China’s cryptocurrency stance^111^.

#### Comparative analysis of neutrality, innovation, and regulation

When comparing the approaches to VAT neutrality and cryptocurrency taxation in the United States and China, distinct regulatory philosophies and economic strategies emerge. The United States, which lacks a VAT system, focuses on capital gains and property taxation to incorporate cryptocurrencies into its fiscal framework, allowing for some level of innovation and market participation while adhering to regulatory requirements. In contrast, China’s centralised approach, characterised by stringent regulations and bans, reflects a different interpretation of neutrality—putting financial control and stability ahead of the market freedoms associated with cryptocurrencies.

Experts such as Varju, Amand, and Grube have discussed the EU’s interpretation of fiscal neutrality, as well as the regulatory stances of the United States and China, highlighting the global challenge of applying VAT neutrality principles to the digital economy so that these are not unduly levied on the businesses^112^. These differences highlight the importance of international dialogue and cooperation in addressing the complexities of taxing digital assets while promoting innovation and economic growth.

#### Conclusion

The United States and China’s opposing views on VAT neutrality and cryptocurrency taxation highlight the larger challenges of integrating digital assets into traditional tax frameworks. While the United States seeks to modify its existing tax laws to accommodate cryptocurrencies in a non-VAT system, China’s approach reflects its broader regulatory and economic goals, even as it considers the implications of digital assets for VAT principles. The evolving debate over VAT neutrality, influenced by global experts and regulatory bodies, continues to shape the international tax landscape in the digital age. Achieving a balance between innovation, regulation, and taxation necessitates continuous adaptation and dialogue among global stakeholders^113^.

### Recommendations for harmonisation of VAT compliance

To improve VAT compliance in the digital economy, a unified VAT framework across EU Member States would reconcile the definition of taxable events, taxable base, and location of supply with the *Skatteverket *verdict. A uniform and dependable environment for digital enterprises encourages compliance and facilitates cross-border transactions^114^. There is a need for new legislation to specifically account for the unique features of Non-Fungible Tokens (NFTs) and address the concerns raised in the European Commission’s Working Paper 1060 regarding the VAT treatment of NFTs—this legislation should include comprehensive guidelines within the VAT framework that cover aspects such as NFT classification, taxable events, and the treatment of related transactions. Concurrently, initiatives for education and awareness are essential; they include actions like webinars, seminars, and training materials to enlighten tax authorities and enterprises on the changing legal landscape, especially with regard to NFTs and cryptocurrencies.

Tax administration may be digitised by investments in digital infrastructure and technology, such as automation tools and sophisticated data analytics, to make it more efficient and error-free. For example, real-time transaction tracking systems and AI-driven compliance checks can be used. As seen by the EU’s collaboration with institutions such as the OECD, international cooperation is necessary to establish worldwide standards for VAT reporting and transparency in the digital economy. The harmonisation of digital asset taxes demonstrates how harmonising worldwide VAT legislation promotes smooth cross-border transactions and supports more effective international corporate operations^115^. Value-added tax (VAT) regulations must be flexible enough to keep up with the rapid improvements in technology in order to allow the digital economy to take advantage of new possibilities and problems—this is shown by the frequent upgrades that facilitate the integration of innovative business methods, such as e‑commerce models or blockchain-based transactions. In order to create effective VAT legislation in the digital domain, business representatives, technical experts, and legal professionals must collaborate. As seen by successful models like the collaborative efforts to produce innovative and fair VAT laws for digital firms, this inclusive approach fosters a deeper understanding of business concerns. technical solutions like blockchain-based transaction tracking and electronic billing systems may help to harmonise VAT compliance^116^. By lowering risks like VAT fraud and enhancing understanding of digital transactions, shared digital tools enable Member States to establish uniform enforcement strategies^117^. Increasing consistency in the application of digital VAT may be accomplished by the European Union (EU) via the organisation of frequent events at which member states share their concerns and best practices^118^. The establishment of a common understanding that permits the implementation of fair VAT enforcement strategies that take into account the intricacies of the digital and crypto services industry is a collaborative effort between governments and organisations from the private sector, such as trade associations and technology companies. Through the incorporation of legal, technological, and procedural concerns, this all-encompassing technique ensures that the legislation governing VAT is enforced in a manner that is both consistent and effective throughout all EU Member States^119^.

The governments may contribute to creating a more standardized, transparent, and flexible environment for VAT compliance in the digital economy by implementing these stated measures—these steps strike a balance between legal clarity and independence to foster an environment where companies may prosper and fulfill their VAT duties in the rapidly evolving digital economy^120^.

### Future research directions on VAT compliance in the digital age

Future research directions in VAT compliance can include the impact of considering industry characteristics, the delineation of digital assets and how well suggested solutions work given technological advances. Lin and Hsieh (2011) emphasise the importance of considering customer characteristics, service interaction characteristics, and employee perspectives when understanding service friendship and compliance in various service settings^121^. These could take the form of mobile payments, which have, according to Wolf (2016), not been addressed by the *Skatteverket *judgement^122^. Alshira’H Alshira’h (2023) emphasises the importance of trust in the government and tax compliance costs in encouraging VAT compliance^123^. Furthermore, Thottoli (2022) investigates the changing role of compliance officers in publicly traded companies in terms of VAT compliance, shedding light on practical implications for future research in this area^124^.

Furthermore, Schoeman et al. (2022) present a quantitative analysis of the relationship between changes in VAT rates and small business compliance behaviour, indicating potential avenues for investigating the impact of policy changes on compliance decisions^125^. Hang and Trinh (2022) identify factors influencing VAT compliance behaviour, providing a framework for future research into the determinants of compliance among corporate taxpayers^126^. The revision of business practices—for instance, the post-Skatteverket VAT legislation for virtual currencies—required large e‑commerce platforms like eBay to overhaul their operating methods, emphasizing the need for EU-wide digital economy norms and collaboration^127^.

Englisch (2022) identified the study of the delineation of digital assets, particularly hybrid tokens, to comprehend the VAT treatment of security tokens^128^. He argues that despite the issuance of security tokens being outside the scope of VAT, issuers could still be entitled to deduct input VAT for related costs, like legal services, if these costs are directly linked to their economic activities and the issuer is a taxable person.

Furthermore, Olsen et al. (2019) discuss the mental accounting of VAT among self-employed business owners, pointing to a potential area of research on how different tax mechanisms influence compliance behaviour^129^. To help the EU’s digital economy be open and fair, there needs to be more unity and guidelines for how VAT is applied right away^130^. Albert and Fadjarenie (2022) argue that early tax education can shape future compliance behaviour, emphasising the importance of educational interventions in instilling tax compliance attitudes at a young age^131^.

Finally, high VAT burdens can make compliance less likely as they increase the burden on businesses, making it more challenging for them to adhere to tax regulations and fulfil their obligations (Vishnuhadevi, 2021)^132^. Additionally, the complexity and high costs of VAT compliance can deter businesses, especially small enterprises, from adhering to VAT regulations, resulting in lower compliance rates (Mascagni et al., 2021)^133^.

To summarise, future research directions in VAT compliance could include exploring the interplay of customer, employee, and service characteristics, understanding the role of trust and compliance costs, investigating the impact of policy changes on compliance behaviour, examining the determinants of compliance among different taxpayer segments, studying the influence of tax education on compliance attitudes, and delving into the mental accounting aspects of VAT in shape.

## Footnotes

1 Tampi J (2021) Non-fungible tokens for trustees. P.C.B. 5:205–211.

2 Case C-264/14 (2015) ECLI:EU:C:2015:498; Kokott, Advocate General (2015) Opinion delivered on 16 July 2015, Case C-264/14, ECLI:EU:C:2015:498. Available via TandF Online: 10.1080/20488432.2015.1096631; Ooi V (2022) Report on the Challenges which Digital Assets Pose for Tax Systems with a Special Focus on Developing Countries. Report Commissioned by the International Tax and Development Cooperation Branch, Financing for Sustainable Development Office, United Nations Department of Economic and Social Affairs, 13. Available at SSRN: https://papers.ssrn.com/sol3/papers.cfm?abstract_id=4395761. Accessed 2 March 2024.

3 Deng H, Chan AFL (2023) The Legal Nature of Cryptocurrency as Property: Accounting and Taxation Implications. Available at SSRN: https://papers.ssrn.com/sol3/papers.cfm?abstract_id=4575634. Accessed 2 March 2024.

4 Beeple (March 2021) Everydays: The First 5000 Days. Available at: https://www.beeple-crap.com/viewing. Accessed 15 February 2024.

5 Akhtar T (2023) TechCrunch Founder’s Apartment to Be Sold as NFT. CoinDesk, May 9. Available at: https://www.coindesk.com/business/2021/05/25/techcrunch-founders-apartment-to-be-sold-as-nft. Accessed 2 March 2024.

6 El Hajjar, Ornina (2022) The regulation of Non-Fungible Tokens (NFTs). PhD diss. Lebanese American University. Available at: https://laur.lau.edu.lb:8443/xmlui/handle/10725/14124. Accessed 2 March 2024.

7 Celaj R (2024) Ethereum Experiment ERC404 Is an NFT-Cryptocurrency Hybrid—and It’s Already Generated $ 87m in Trades. DL News, February 8. Available at: https://www.dlnews.com/articles/defi/ethereum-token-experiment-erc404-nft-cryptocurrency-hybrid/. Accessed 3 March 2024.

8 El Hajjar, Ornina (2022) The regulation of Non-Fungible Tokens (NFTs). PhD diss., Lebanese American University. Available at: https://laur.lau.edu.lb:8443/xmlui/handle/10725/14124. Accessed 2 March 2024.

9 Taxation and Customs Union (n. d.) VAT Expert Group. Available at: https://taxation-customs.ec.europa.eu/taxation-1/value-added-tax-vat/vat-expert-group_en. Accessed 22 February 2024.

10 Case C-264/14 (2015) ECLI:EU:C:2015:498.

11 The European Union (n. d.) The European Union’s Common System of Value Added Tax (VAT). EUR-Lex. Available at: https://eur-lex.europa.eu/EN/legal-content/summary/the-european-union-s-common-system-of-value-added-tax-vat.html. Accessed 23 February 2024.

12 Case C-264/14 (2015) ECLI:EU:C:2015:498.

13 Huertas M, Hikl A (2022) Non-fungible tokens (NFTs)—new opportunities but do they need new EU regulations? J.I.B.L.R. 37(4):132–144.

14 Case C-264/14, [2015] ECLI:EU:C:2015:498.

15 Huertas M, Hikl A (2022) Non-fungible tokens (NFTs)—new opportunities but do they need new EU regulations? J.I.B.L.R. 37(4):132–144.

16 De Mooij, Hebous S, Keen M KBA (2023) Taxing Cryptocurrencies. IMF, June 30. Available at: https://www.imf.org/en/Publications/WP/Issues/2023/06/30/Taxing-Cryptocurrencies-535510; European Commission (2023) Working Paper 1060—Commission Question on Non-Fungible Tokens. Available at: https://circabc.europa.eu/ui/group/cb1eaff7-eedd-413d-ab88-94f761f9773b/library/7d1ef2eb-b820-4866-a155-785e2373fb80/details. Accessed 23 February 2024.

17 Asquith R (2020) The gig and sharing economies: millions of new entrepreneurs; billions in lost VAT. B.T.R. 1:5–22.

18 Kokott, Advocate General (2015) Opinion delivered on 16 July 2015, Case C-264/14. ECLI:EU:C:2015:498. Available via TandF Online: 10.1080/20488432.2015.1096631.

19 Kokott, Advocate General (2015) Opinion delivered on 16 July 2015, Case C-264/14. ECLI:EU:C:2015:498. Available via TandF Online: 10.1080/20488432.2015.1096631.

20 Bauer M, Erixon F (2016) Competition, Growth and Regulatory Heterogeneity in Europe’s Digital Economy. ECIPE Working Paper 2. Available at: https://ecipe.org/wp-content/uploads/2016/04/Competition-Growth-and-Regulatory-Heterogeneity-in-Europe%E2%80%99s-Digital-Economy-final1.pdf. Accessed 25 February 2024; Cruz Cano M (2019) Amazon: OECD should adopt VAT principles to simplify digital tax plan. International Tax Review. London. Available at: https://www.proquest.com/openview/1a3299cfaca7936d0b5ca040dc47eef3/1?pq-origsite=gscholar&cbl=30282 Accessed 25 February 2024; Dashe DN, Asada D (2023) Multi-Lateral Global and Unilateral Initiatives, Antidotes for Effective Regulation and Administration of the Taxation of Internet Transactions. IJLDLR 12:64. Available at: https://heinonline.org/hol-cgi-bin/get_pdf.cgi?handle=hein.journals/intjadr12&section=8. Accessed 25 February 2024.

21 Kokott, Advocate General (2015) Opinion delivered on 16 July 2015, Case C-264/14. ECLI:EU:C:2015:498. Available via TandF Online: 10.1080/20488432.2015.1096631.

22 Künnapas K, Bernabeu BP, Kuum K, Pungas KO (2023) Taxes on the Digital Economy. In: Troitiño DR, Kerikmäe T, Hamuľák O (eds) Digital Development of the European Union. Springer, Cham. 10.1007/978-3-031-27312-4_7. Accessed 2 March 2024; Santana S (2016) Regulating Virtual Currency: Case C-264/14 Skatteverket v. David Hedqvist [2015] ECLI: EU: C: 2015: 718. N. Ir. Legal Q. 67:109. Available at: https://nilq.qub.ac.uk/index.php/nilq/article/download/99/70/127. Accessed 3 March 2024.

23 “Buying bitcoins exempt from VAT” (2015) EU Focus 338:26.

24 EUR-Lex (n. d.) Legal Documents. Available at: https://eur-lex.europa.eu/legal-content/EN/ALL/?uri=celex%3A32006L0112. Accessed 20 February 2024.

25 Nivedha S (2022) Progression of Cryptocurrency: A Taxation Concern. International Journal of Law Management & Humanities 5(1):273–xxx.

26 Asquith R, “Bitcoin Wins EU VAT Case—Avalara” (Avalara, Inc.). Available at: https://www.avalara.com/vatlive/en/vat-news/bitcoin-wins-eu-vat-case.html. Accessed 16 February 2024.

27 “62014CC0264.” Available at: https://eur-lex.europa.eu/legal-content/EN/TXT/HTML/?uri=CELEX:62014CC0264&from=EN . Accessed 16 February 2024.

28 European Commission Group on the Future of VAT (2014) Working Paper 049 VAT Treatment of Bitcoin. Available at: https://circabc.europa.eu/sd/a/4adc83f8-a7ab-48ee-b907-468459c0dad7/49%20-%20VAT%20treatment%20of%20Bitcoin.pdf. Accessed 16 February 2024.

29 Tuchtfeld E, “The ECJ’s First Bitcoin Decision” (Völkerrechtsblog, December 9, 2020). Available at: 10.17176/20170925-174324. Accessed 16 February 2024.

30 Kane J (2016) VAT Directive—exemptions—bitcoin virtual currency. C.L. Pract. 23(4):116–117.

31 Santana S (2016) Regulating Virtual Currency: Case C-264/14 Skatteverket v. David Hedqvist [2015] ECLI: EU: C: 2015: 718. N. Ir. Legal Q. 67:109. Available at: https://nilq.qub.ac.uk/index.php/nilq/article/download/99/70/127. Accessed 3 March 2024.

32 Kollmann J (2019) The VAT Treatment of Cryptocurrencies. EC Tax Review 28(3):164–170.

33 Bruins K, van de Berg R, van Goor E (2015) The Hedqvist ruling and the impact of EU VAT on Bitcoin. E.F.P.L. & P. 9(12):6–7.

34 Tuchtfeld E, “The ECJ’s First Bitcoin Decision” (Völkerrechtsblog, December 9, 2020). Available at: 10.17176/20170925-174324. Accessed 16 February 2024.

35 Nahorniak I, Leonova K, Skorokhod V (2016) Cryptocurrency in the Context of Development of Digital Single Market in European Union. InterEULawEast: Journal for International and European Law, Economics and Market Integrations 3(1):107–124.

36 Santana S (2016) Regulating Virtual Currency: Case C-264/14 Skatteverket v. David Hedqvist [2015] ECLI: EU: C: 2015: 718. N. Ir. Legal Q. 67:109. Available at: https://nilq.qub.ac.uk/index.php/nilq/article/download/99/70/127. Accessed 3 March 2024.

37 European Commission Group on the Future of VAT (2014) Working Paper 049 VAT Treatment of Bitcoin. Available at: https://circabc.europa.eu/sd/a/4adc83f8-a7ab-48ee-b907-468459c0dad7/49%20-%20VAT%20treatment%20of%20Bitcoin.pdf. Accessed 16 March 2024.

38 Aparicio MG (2018) Taxation of the Bitcoin on the Value Added Tax: Particularly, Judgment of the European Court in Case C 264/14 (22 October 2015). In Cluj Tax Forum 1(3):5–12. Editura Hamangiu SRL. Available at: https://www.ceeol.com/search/article-detail?id=814534.

39 Veerpalu A (2018) Decentralised Technology and Technology Neutrality in Legal Rules: An Analysis of and. Baltic Journal of Law & Politics 11(2):61–94. Available at: https://sciendo.com/article/10.2478/bjlp-2018-0011.

40 Lee J, Van de Looverbosch M (2021) Property and data: a confused relationship. In Data Governance in AI, FinTech and RegTech: Law and Regulation in the Financial Sector. Cheltenham, Edward Elgar. Available at: https://papers.ssrn.com/sol3/papers.cfm?abstract_id=3995492.

41 Santana S (2016) Regulating Virtual Currency: Case C-264/14 Skatteverket v. David Hedqvist [2015] ECLI: EU: C: 2015: 718. N. Ir. Legal Q. 67:109. Available at: https://nilq.qub.ac.uk/index.php/nilq/article/download/99/70/127. Accessed 3 March 2024.

42 Daniel P (2021) Relations between the principle of neutrality and elements of value added tax structure. Financial Internet Quarterly 17.3:56–63. Available at: https://sciendo.com/pdf/10.2478/fiqf-2021-0019. Accessed 3 March 2024.

43 European Commission Group on the Future of VAT (2014) Working Paper 049 VAT Treatment of Bitcoin. Available at: https://circabc.europa.eu/sd/a/4adc83f8-a7ab-48ee-b907-468459c0dad7/49%20-%20VAT%20treatment%20of%20Bitcoin.pdf. Accessed 16 March 2024.

44 Asquith R, “Bitcoin Wins EU VAT Case—Avalara” (Avalara, Inc.). Available at: https://www.avalara.com/vatlive/en/vat-news/bitcoin-wins-eu-vat-case.html. Accessed 16 February 2024.

45 EUR-Lex (n. d.) Legal Documents. Available at: https://eur-lex.europa.eu/legal-content/EN/ALL/?uri=celex%3A32006L0112. Accessed: 20 February 2024.

46 Granton Advertising BV v Inspecteur van de Belastingdienst Haaglanden/kantoor Den Haag (C-461/12), [2014] EU:C:2014:1745, [2014] S.T.C. 2375, [2014] 6 WLUK 336, [2014] B.V.C. 31, [2014] S.T.I. 2113.

47 Le Rayon d’Or Sarl v Ministre de l’Economie et des Finances (C-151/13), [2014] EU:C:2014:185.

48 First National Bank of Chicago v Customs and Excise Commissioners (C172/96), [1998] EU:C:1998:354.

49 Squires L, Bantock F (2015) The VAT briefing for November. Tax J. 1284:16–17. Available at: https://www.taxjournal.com/articles/vat-briefing-november-05112015. Accessed 15 February 2024.

50 Sankey J (2016) VAT treatment of Bitcoin—a proxy for other cryptocurrencies. Euro. L.R. 6:223–228.

51 Srokosz W, Kopyscianski T (2015) Legal and Economic Analysis of the Cryptocurrencies Impact on the Financial System Stability. Journal of Teaching and Education 04(02):619–627. Available at: https://www.universitypublications.net/jte/0402/pdf/F5N180.pdf. Accessed 2 February 2024.

52 Badea L, Mungiu-Pupӑzan MC (2021) The Economic and Environmental Impact of Bitcoin. IEEE Access 9:48091–48104. 10.1109/ACCESS.2021.3068636. Available at: https://ieeexplore.ieee.org/abstract/document/9385063. Accessed 2 February 2024.

53 Musleh AS, Yao G, Muyeen SM (2019) Blockchain Applications in Smart Grid—Review and Frameworks. IEEE Access 7:86746–86757. 10.1109/access.2019.2920682. Accessed 3 March 2024.

54 Shrivastava A, Khamroi A (2017) Smart contracts—the way forward. Available at: https://papers.ssrn.com/sol3/papers.cfm?abstract_id=3846176. Accessed 3 March 2024.

55 Sakız B, Gencer AH (2021) Blockchain Beyond Cryptocurrency: Non-Fungible Tokens. International Conference on Eurasian Economies. 10.36880/c13.02527. Accessed 3 March 2024.

56 Elliott B (2022) Evolving technologies: VAT, cryptoassets and the metaverse. Tax J. 1584:17–19. Available at: https://files.pumptax.com/wp-content/uploads/2022/08/03102815/BENTJ_Elliott.pdf. Accessed 15 March 2024.

57 de la Feria R, Krever R (2013) Ending VAT Exemptions: Towards a Post-Modern VAT. In: de la Feria R (ed) VAT Exemptions: Consequences and Design Alternatives, pp 3–36. Wolters Kluwer. Available at SSRN: https://ssrn.com/abstract=2273111. Accessed 3 March 2024.

58 Ooi V (2022) Report on the Challenges which Digital Assets Pose for Tax Systems with a Special Focus on Developing Countries. Report Commissioned by the International Tax and Development Cooperation Branch, Financing for Sustainable Development Office, United Nations Department of Economic and Social Affairs, 13. Available at SSRN: https://papers.ssrn.com/sol3/papers.cfm?abstract_id=4395761.

59 “Tax Treatment of Cryptoassets” (2021) Global Digital Finance (GDF), February. Available at: https://www.gdf.io/wp-content/uploads/2021/02/GDF-Tax-Treatment-of-Cryptoassets.pdf. Accessed 15 February 2024.

60 “Tax Treatment of Cryptoassets” (2021) Global Digital Finance (GDF), February. Available at: https://www.gdf.io/wp-content/uploads/2021/02/GDF-Tax-Treatment-of-Cryptoassets.pdf. Accessed 15 February 2024.

61 Elliott B (2022) Evolving technologies: VAT, cryptoassets and the metaverse. Tax J. 1584:17–19. Available at: https://files.pumptax.com/wp-content/uploads/2022/08/03102815/BENTJ_Elliott.pdf. Accessed 15 March 2024.

62 Van Doesum A, Van Kesteren H (2016) ‘1.4 Basic Mechanism of the EU VAT System’. In: Fundamentals of EU VAT Law. Wolters Kluwer. Available at: https://books.google.de/books?id=YoyWDwAAQBAJ. Accessed 16 March 2024.

63 Müller T (2022) Geld ohne Staat – Zur Regulierung von Kryptoassets in der Europäischen Union. ÖZW 2022;150: Heft 4 v. 15.09.2022.

64 BMF (2017) Zur steuerlichen Behandlung von Kryptowährungen (virtuellen Währungen). RdW 2017/425; RdW 2017;580: Heft 8 v. 23.8.2017.

65 Kadikov A (2015) International taxation of cross-border digital commerce. PhD diss., University of Oxford. Available at: https://ora.ox.ac.uk/objects/uuid:ea6c6f2e-c65f-4fa5-945a-22eb71e12667. Accessed 16 March 2024.

66 Boeve R, Arrieta R (2016) European VAT case highlights growing use of Bitcoin. I.T. Rev. Jun:34–35. Available at: https://www.proquest.com/docview/1799508300. Accessed 16 March 2024.

67 *Vega International Car Transport and Logistic – Trading GmbH v Dyrektor Izby Skarbowej w Warszawie (C- 235/18), [2019] EU:C:2019:412.*

68 “EUR-Lex—62019CJ0422—EN—EUR-Lex.” Available at: https://eur-lex.europa.eu/legal-content/EN/TXT/?uri=CELEX%3A62019CJ0422. Accessed 16 March 2024.

69 Dietrich v Hessischer Rundfunk (C-422/19), [2021] WL 241081.

70 Taylor D (2021) VAT, fintech and the payment transfer exemption. Tax J. 1525:26–27. Available at: https://taxscape.deloitte.com/article/vat--fintech-and-the-payment-transfer-exemption.aspx. Accessed 15 March 2024

71 Court of Justice of the European Union Press and Information (2015) Court of Justice of the European Union PRESS RELEASE No 128/15: The Exchange of Traditional Currencies for Units of the ‘Bitcoin’ Virtual Currency Is Exempt from VAT. Available at: https://curia.europa.eu/jcms/upload/docs/application/pdf/2015-10/cp150128en.pdf. Accessed 16 March 2024.

72 Chesham M (2022) Exchanging coins for voucher. In VAT and Financial Services (4th ed.), p. 20. Spiramus Press Ltd. Available at: https://books.google.de/books?id=5yx7EAAAQBAJ. Accessed 16 March 2024.

73 Coinstar Ltd v Revenue and Customs Commissioners, [2016] WL 04591795.

74 “Decision Detail” (Bundesfinanzhof, February 27, 2024). Available at: https://www.bundesfinanzhof.de/en/entscheidungen/entscheidungen-online/decision-detail/STRE202310036/. Accessed 15 March 2024.

75 Deutschland P (2023) EuGH-Vorlage Zur Besteuerung von Gutscheinen. PwC-Blogs. Available at: https://blogs.pwc.de/de/steuern-und-recht/article/235771/eugh-vorlage-zur-besteuerung-von-gutscheinen. Accessed 15 March 2024.

76 “TaxScape | Deloitte” (Target Group Ltd—Court of Appeal Judgment). Available at: https://taxscape.deloitte.com/article/target-group-ltd—-court-of-appeal-judgment.aspx. Accessed 16 March 2024.

77 “Europe: United Kingdom Supreme Court Judgment in Target Case—Taxpayer Loss.” Available at: https://insightplus.bakermckenzie.com/bm/international-commercial-trade/europe-united-kingdom-supreme-court-judgment-in-target-case-taxpayer-loss/. Accessed 16 March 2024.

78 Palmér G (2017) Why do regulatory practices towards Uber diverge in the globalized economy? Comparing regulatory responses and attitudes towards Uber in the U.S. and Sweden. Uppsala Universitet. Available at: https://www.diva-portal.org/smash/get/diva2:1105482/FULLTEXT01.pdf.

79 Target Group Ltd v Revenue and Customs Commissioners, [2021] WL 02905821.

80 Marinc M, van de Berg R (2015) European Union: decision on landmark case regarding the VAT treatment of bitcoin. Euro. T.S. Nov:37.

81 Nahorniak I, Leonova K, Skorokhod V (2016) Cryptocurrency in the Context of Development of Digital Single Market in European Union. InterEULawEast: J Int’l & Eur L, Econ & Market Integrations 3:107; Wolf R (2016) Skatteverket v. Hedqvist (CJEU). International Legal Materials 55(4):720–730. Available at: https://heinonline.org/hol-cgi-bin/get_pdf.cgi?handle=hein.journals/intlm55&section=41. Accessed 5 March 2024.

82 Celaj R (2024) Ethereum Experiment ERC404 Is an NFT-Cryptocurrency Hybrid—and It’s Already Generated $ 87m in Trades. DL News, February 8. Available at: https://www.dlnews.com/articles/defi/ethereum-token-experiment-erc404-nft-cryptocurrency-hybrid/. Accessed 3 March 2024.

83 Santana S (2016) Regulating Virtual Currency: Case C-264/14 Skatteverket v. David Hedqvist [2015] ECLI: EU: C: 2015: 718. N. Ir. Legal Q. 67:109. Available at: https://nilq.qub.ac.uk/index.php/nilq/article/download/99/70/127. Accessed 3 March 2024.

84 Houben R (2016) The CJEU’s view of whether Bitcoins are a currency: a Belgian perspective. I.C.C.L.R. 27(3):61–64.

85 Aksoy PC (2023) The Regulation of NFTs: Much Ado About Nothing?. Business Law Review 44(4). Available at: https://kluwerlawonline.com/journalarticle/Business+Law+Review/44.4/BULA2023016. Accessed 18 February 2024.

86 Nahorniak I, Leonova K, Skorokhod V (2016) Cryptocurrency in the Context of Development of Digital Single Market in European Union. InterEULawEast: J Int’l & Eur L, Econ & Market Integrations 3:107.

87 Cappai M (2023) The role of private and public regulation in the case study of crypto-assets: The Italian move towards participatory regulation. Computer Law & Security Review 49:105831. Available at: https://www.sciencedirect.com/journal/computer-law-and-security-review/vol/49/suppl/C. Accessed 1 March 2024.

88 Deng H, Chan AFL (2023) The Legal Nature of Cryptocurrency as Property: Accounting and Taxation Implications. Available at SSRN: https://papers.ssrn.com/sol3/papers.cfm?abstract_id=4575634. Accessed 2 March 2024.

89 Houben R (2016) The CJEU’s view of whether Bitcoins are a currency: a Belgian perspective. I.C.C.L.R. 27(3):61–64; Ooi V (2022) Report on the Challenges which Digital Assets Pose for Tax Systems with a Special Focus on Developing Countries. Report Commissioned by the International Tax and Development Cooperation Branch, Financing for Sustainable Development Office, United Nations Department of Economic and Social Affairs, 13. Available at SSRN: https://papers.ssrn.com/sol3/papers.cfm?abstract_id=4395761. Accessed 5 February 2024.

90 Beukes W (2020) Value-added tax: a critical analysis of input tax in respect of share issue costs. PhD diss., Stellenbosch University. Available at: https://scholar.sun.ac.za/handle/10019.1/108332. Accessed 5 March 2024.

91 Value Added Tax Committee (2021) Working Paper No 1060: Initial VAT reflections on non-fungible tokens. Articles 2, 9, 47 and 58.

92 Ooi V (2022) Report on the Challenges which Digital Assets Pose for Tax Systems with a Special Focus on Developing Countries. Report Commissioned by the International Tax and Development Cooperation Branch, Financing for Sustainable Development Office, United Nations Department of Economic and Social Affairs, 13.

93 Wolf R “introductory note to Skatteverket v. Hedqvist (CJEU)”. Available at: https://heinonline.org/hol-cgi-bin/get_pdf.cgi?handle=hein.journals/intlm55&section=41.

94 Aksoy PC (2023) The Regulation of NFTs: Much Ado About Nothing?. Business Law Review 44(4). Available at: https://kluwerlawonline.com/journalarticle/Business+Law+Review/44.4/BULA2023016. Accessed 10 March 2024.

95 Zilioli C (2020) Crypto-assets: legal characterisation and challenges under private law. E.L. Rev. 45(2):251–266.

96 Elliott B (2022) Evolving technologies: VAT, cryptoassets and the metaverse. Tax J. 1584:17–19. https://files.pumptax.com/wp-content/uploads/2022/08/03102815/BENTJ_Elliott.pdf. Accessed 15 March 2024; Shambhavee HM, Sujee P (2021) Sui Generis Law regarding Cryptocurrency; a Need of the Hour. Supremo Amicus 27:[159].

97 Juanpere BA (2019) Action Plan on VAT: Creating a Single VAT Area in the EU. Eurasian Journal of Economics and Finance 7(1):1–14. Available at: https://eurasianpublications.com/wp-content/uploads/2021/02/EJEF-7.1.1.pdf. Accessed 7 March 2024.

98 Neagu A (2021) Cryptocurrency Regulation and Taxation in Europe. Cluj Tax FJ:286.

99 Zilioli C (2020) Crypto-assets: legal characterisation and challenges under private law. E.L. Rev. 45(2):251–266.

100 Zilioli C (2020) Crypto-assets: legal characterisation and challenges under private law. E.L. Rev. 45(2):251–266.

101 Merkx M (2019) VAT and Blockchain: Challenges and Opportunities Ahead. EC Tax Review 28(2):83–89. Available at: https://kluwerlawonline.com/journalarticle/EC+Tax+Review/28.2/ECTA2019011. Accessed 15 March 2024.

102 Danescu E (2020) Taxing intangible assets: issues and challenges for a digital Europe. Internet Histories 4(2):196–216.

103 Voronin VN (2021) Issues of Strategic Legal Regulation of the Implementation of Digital Technologies in Transport. Siberian Law Review 18:128–137. Available at: https://scholar.archive.org/work/2ywminp7knasdctsv66e4vzmai/access/wayback/https://www.siberianlawreview.ru/jour/article/download/1651/1488. Accessed 20 February 2024; West JK (2019) An introduction to online platforms and their role in the digital transformation. Available at SSRN: https://papers.ssrn.com/sol3/papers.cfm?abstract_id=4669281. Accessed 20 February 2024.

104 Ooi V (2022) Report on the Challenges which Digital Assets Pose for Tax Systems with a Special Focus on Developing Countries. Report Commissioned by the International Tax and Development Cooperation Branch, Financing for Sustainable Development Office, United Nations Department of Economic and Social Affairs, 13. Available at: https://papers.ssrn.com/sol3/papers.cfm?abstract_id=4395761.

105 Andersson E (2022) A Comparative Analysis of the Taxation of Crypto Currencies. Available at: https://www.diva-portal.org/smash/get/diva2:1664768/FULLTEXT01.pdf.

106 West JK (2019) An introduction to online platforms and their role in the digital transformation. Available at SSRN: https://papers.ssrn.com/sol3/papers.cfm?abstract_id=4669281. Accessed 20 February 2024.

107 Lee D, Kim D, Borcherding T (2013) Tax structure and government spending: does the value-added tax increase the size of government? National Tax Journal 66(3):541–569. 10.17310/ntj.2013.3.02. Accessed 14 February 2024.

108 Internal Revenue Service. Frequently Asked Questions on Virtual Currency Transactions. Available at: https://www.irs.gov/individuals/international-taxpayers/frequently-asked-questions-on-virtual-currency-transactions. Accessed 14 February 2024.

109 Lambert EE (2015–2016) The Internal Revenue Service and Bitcoin: A Taxing Relationship. Va. Tax Rev. 35:88. Available at: https://heinonline.org/HOL/LandingPage?handle=hein.journals/vrgtr35&div=6&id=&page=. Accessed 14 February 2024.

110 Li J, Wang X (2020) Does VAT have higher tax compliance than a turnover tax? Evidence from China. Int Tax Public Finance 27:280–311. 10.1007/s10797-019-09567-4. Accessed 15 March 2024.

111 South China Morning Post (2024) Shanghai Explainer on Taxation of Digital Currency Transaction Drives Speculation on Potential Easing of China’s Crypto Ban. January 8. Available at: https://finance.yahoo.com/news/shanghai-explainer-taxation-digital-currency-093000727.html. Accessed 3 March 2024.

112 Brysland G (n. d.) Fiscal neutrality: Foreign ghost in our GST machine? eJournal of Tax Research 18(1):193–301.

113 Brysland G (n. d.) Fiscal neutrality: Foreign ghost in our GST machine? eJournal of Tax Research 18(1):193–301.

114 Beukes W (2020) Value-added tax: a critical analysis of input tax in respect of share issue costs. PhD diss., Stellenbosch University. Available at: https://scholar.sun.ac.za/handle/10019.1/108332. Accessed 10 March 2024.

115 Asquith R (2015) VAT exemption boosts Bitcoin. Accountancy 155(1468):31; Wolf R (2016) INTRODUCTORY NOTE TO SKATTEVERKET V. HEDQVIST (CJEU). Available at: https://heinonline.org/hol-cgi-bin/get_pdf.cgi?handle=hein.journals/intlm55&section=41. Accessed 10 March 2024.

116 Deng H, Chan AFL (2023) The Legal Nature of Cryptocurrency as Property: Accounting and Taxation Implications. Available at SSRN: https://papers.ssrn.com/sol3/papers.cfm?abstract_id=4575634. Accessed 10 March 2024.

117 Ooi V (2022) Report on the Challenges which Digital Assets Pose for Tax Systems with a Special Focus on Developing Countries. Report Commissioned by the International Tax and Development Cooperation Branch, Financing for Sustainable Development Office, United Nations Department of Economic and Social Affairs, 13. Available at: https://papers.ssrn.com/sol3/papers.cfm?abstract_id=4395761. Accessed 10 March 2024.

118 West JK (2019) An introduction to online platforms and their role in the digital transformation. Available at SSRN 4669281. Available at SSRN: https://papers.ssrn.com/sol3/papers.cfm?abstract_id=4669281. Accessed 10 March 2024.

119 Deng H, Chan AFL (2023) The Legal Nature of Cryptocurrency as Property: Accounting and Taxation Implications. Available at SSRN: https://papers.ssrn.com/sol3/papers.cfm?abstract_id=4575634. Accessed 10 March 2024.

120 Elgaard KKE (2020) Recent Norwegian Supreme Court case law on VAT grouping and VAT deduction of transaction costs related to the sale and purchase of shares from an EU and a Danish perspective—Part two. Skatterett 39(2):101–135.

121 Lin J, Hsieh C (2011) Modeling service friendship and customer compliance in high-contact service relationships. Journal of Service Management 22(5):607–631. 10.1108/09564231111174979. Available at: https://www.emerald.com/insight/content/doi/10.1108/09564231111174979/full/html. Accessed 9 March 2024.

122 Wolf R (2016) Virtual Currencies, M‑Payments and VAT: Ready for the Future? Palgrave Macmillan UK eBooks. Available at: https://link.springer.com/chapter/10.1057/978-1-137-57512-8_10. Accessed 9 March 2024.

123 Alshira’h AF (2023) How can value added tax compliance be incentivized? an experimental examination of trust in government and tax compliance costs. Journal of Anti Money Laundering Control 27(1):191–208. 10.1108/jmlc-01-2023-0009. Available at: https://www.emerald.com/insight/content/doi/10.1108/JMLC-01-2023-0009/full/html. Accessed 13 March 2024.

124 Thottoli MM (2022) A study on listed companies’ compliance with value-added tax: the evolving role of compliance officer in Oman. Public Administration and Policy: An Asia-Pacific Journal 25(1):89–98. 10.1108/pap-02-2021-0017. Available at: https://www.emerald.com/insight/content/doi/10.1108/PAP-02-2021-0017/full/html. Accessed 13 March 2024.

125 Schoeman AHA, Evans C, Preez Hd (2022) To register or not to register for value-added tax? how tax rate changes can influence the decisions of small businesses in South Africa. Meditari Accountancy Research 30(7):213–236. 10.1108/medar-05-2021-1309. Available at: https://www.emerald.com/insight/content/doi/10.1108/MEDAR-05-2021-1309/full/html. Accessed 13 March 2024.

126 Hằng NT, Trinh TTN (2022) Factors affecting value added tax compliance behavior of enterprise: a study of the tax office of Binh Tan District, Ho Chi Minh City. Journal of Science and Technology Issue on Information and Communications Technology. 10.31130/ud-j st.2022.226e. Available at: https://jst-ud.vn/jst-ud/article/view/7871. Accessed 13 March 2024.

127 “Grundsatz Zu Virtuellen Währungen” (eBay). Available at: https://www.ebay.de/help/policies/prohibited-restricted-items/grundsatz-zu-kryptowhrungen?id=5044. Accessed 13 March 2024.

128 Englisch J (2022) VAT Goes Virtual: Security Tokens. EC Tax Review 31(5):232–237. Available at SSRN: https://papers.ssrn.com/sol3/papers.cfm?abstract_id=4209502. Accessed 15 March 2024.

129 Olsen J, Kasper M, Muehlbacher S, Kirchler E (2019) Mental accounting of income tax and value added tax among self-employed business owners. Journal of Economic Psychology 70:125–139. 10.1016/j.joep.2018.12.007. Available at: https://www.sciencedirect.com/science/article/abs/pii/S0167487018301508?via%3Dihub. Accessed 13 March 2024.

130 Value Added Tax Committee (2021) Working Paper No 1060: Initial VAT reflections on non-fungible tokens.

131 Albert A, Fadjarenie RA (2022) Early tax education: could it change the future compliance behavior?. International Journal of Evaluation and Research in Education (IJERE) 11(4):1912. 10.11591/ijere.v11i4.22241. Available at: https://ijere.iaescore.com/index.php/IJERE/article/view/22241. Accessed 13 March 2024.

132 Vishnuhadevi S (2021) Administrative and compliance costs of value added tax (vat): a review. Review of Development and Change 26(2):179–206. 10.1177/09722661211058807. Available at: https://journals.sagepub.com/doi/10.1177/09722661211058807. Accessed 13 March 2024.

133 Mascagni G, Dom R, Santoro F (2021) The VAT in practice: equity, enforcement and complexity. 10.19088/ictd.2021.002. Available at: https://opendocs.ids.ac.uk/opendocs/handle/20.500.12413/15915. Accessed 13 March 2024.

